# Tribal-Led Commercial Tobacco Prevention: Outcomes from the California American Indian Tobacco Initiative Evaluation

**DOI:** 10.1007/s40615-025-02643-y

**Published:** 2025-09-15

**Authors:** Nathaniel J. Gonzalez, Lauren Nippoldt, Kelly Nielsen, Chag Lowry, Alexxa R. Casanova, Georgia C. Kovacs, Wael K. Al-Delaimy

**Affiliations:** 1 Center for Research and Evaluation, University of California San Diego, San Diego, CA USA; 2 Herbert Wertheim School of Public Health & Human Longevity Science, University of California San Diego, San Diego, CA USA

**Keywords:** Tobacco prevention and control, Indigenous, American Indian/Alaskan Native, Community partnerships, Evaluation, Cultural competence

## Abstract

Commercial tobacco-related health disparities persist for American Indian and Alaska Natives in the USA despite widespread reductions in commercial tobacco use in the general population. Addressing these disparities requires a culturally relevant approach but the diversity of Tribal communities, including historical, cultural, social, and economic differences, can make uniform public health interventions that are culturally relevant challenging to design. The California Tribal Initiative to Reduce Tobacco-Related Disparities, launched in 2019 by the California Department of Public Health and the California Tobacco Prevention Program, uniquely addresses these disparities by directly funding Tribal governments and American Indian Serving Community Organizations to develop tailored commercial tobacco prevention projects that will work for them. This article presents the results from the California American Indian Tobacco Initiative Evaluation, tasked with assessing the initiative’s success. The study used a longitudinal and mixed-methods approach between 2019 and 2024, employing surveys, key informant interviews, and talking circles to assess the initiative through progress in three areas: enhancing community engagement, advancing and adhering to commercial tobacco prevention policies, and building Tribal capacities to sustain these efforts. Findings indicate significant improvements across all areas, underscoring the efficacy of empowering Tribes to lead health interventions. This approach not only advances public health but also fosters greater trust and collaboration between Tribes and public health entities.

Commercial tobacco remains one of the leading causes of illness and death in the USA [1]. Despite substantial progress by the California Department of Public Health (CDPH) and the California Tobacco Prevention Program (CTPP) in reducing smoking rates, with some of the nation’s lowest at 6.0% among adults and 1.2% among youth, significant disparities persist in California [2]. Commercial tobacco usage rates among California American Indians are significantly higher compared to California’s average commercial tobacco use rate and compared to other racial and ethnic groups [2]. Diseases linked to commercial tobacco use including cardiovascular disease, cancer, and diabetes are leading causes of death for American Indians and Alaska Natives (AI/AN) nationally, with historically higher death rates compared to other racial and ethnic groups [3–5].

Addressing these disparities requires additional efforts to support commercial tobacco prevention strategies that are locally resonant. As Blue Bird Jernigan and coauthors argue, to overcome mistrust borne of a long history of racism in medical research with AI/AN communities, projects need to work closely with Tribal communities to ensure they are “culturally centered, a community priority, and perceived by community members to hold real value and promise for improving Native health” [6]. Such culturally relevant programming respects the sovereignty of Tribes, helps restore relationships of trust between Tribes and researchers, and has furthermore shown promise in curbing commercial tobacco use disparities [7]. However, the diversity within California’s Tribal communities, including historical, cultural, social, and economic differences, can make uniform public health interventions that are culturally relevant challenging to design.

Launched in 2019 and funded by the CDPH/CTPP, the California Tribal Initiative to Reduce Tobacco-Related Disparities, also called the American Indian Initiative (AII), tackles this challenge by directly funding Tribal governments and American Indian Serving Community Organizations (AISCOs) to develop and implement programming that works for them. This innovative funding approach not only respects Tribal sovereignty but also elevates the unique cultural strengths of each community to develop effective tobacco control and prevention strategies. The California American Indian Tobacco Initiative Evaluation (CAITIE) was tasked with evaluating the success of this initiative. To do so, the evaluation used data from surveys, interviews, and talking circles to measure the initiative’s success in three areas: (1) enhancing community engagement with commercial tobacco prevention programming, (2) passing and improving community adherence to commercial tobacco prevention policies, and (3) building Tribal capacity to address commercial tobacco use disparities. The aim was not to evaluate the Tribes or specific projects but rather to evaluate the AII with the goal of improving how the state supports commercial tobacco prevention in California Tribal communities. The results of the evaluation, outlined in this article, indicate significant improvements over time across all three areas. These findings underscore the effectiveness of directly funding Tribal governments and organizations to develop culturally relevant programming.

## Methodology

The evaluation research was conducted between 2019 and 2024, with data collection occurring between July 2020 and January 2024. It employed a mixed-methods recurrent cross-sectional design using a longitudinal qualitative research (LQR) approach; the analysis explored themes over time through data collected from the same population multiple times [8, 9]. The specific methods selected for the evaluation include both qualitative (key informant interviews and talking circles) and quantitative (key informant online surveys). Participants were surveyed and interviewed twice as part of this project, with an average time of two years between baseline and follow-up.

Outreach to American Indian Initiative projects and Tribal community members was a major task led by a dedicated staff. These staff were members of the CAITIE research team and California Natives, one of whom is descended from multiple Northern California Tribal cultures, and the other from a Tribe in Southern California. Both team members had relationships with Tribal communities they could rely on to facilitate communication within Tribal families and governments. These relationships, whether directly personal or through social or cultural ties, ensured that outreach was done in a way that was sensitive to the shared history and, where possible, the specific contexts of participating Tribes.

To further ensure a culturally sensitive approach to engaging Tribes, the project directors (PD) of the American Indian Initiative-funded projects identified staff and community members who would be most appropriate for participating in the key informant surveys, interviews, and talking circles, in accordance with the memorandum of understanding signed between Tribal governments, AISCOs, and the State of California. This gave Tribes greater control over the research process. Project directors were encouraged to identify key informants that were over 18 years old, lived in the community, and were knowledgeable about commercial tobacco prevention work in their community.

### Surveys

The key informant surveys included Likert-scale questions on commercial tobacco prevention policies, community awareness of health risks, and the prevalence of commercial tobacco sales. Participants also estimated community tobacco use rates and provided qualitative feedback on project effectiveness. Established scales were utilized where possible, including the Project Sustainability Assessment Tool (PSAT) [10]. Demographic information was collected to identify potential confounders.

A total of 142 survey responses were collected, including 86 at baseline and 56 at follow-up (Table 1). Respondents were predominantly female (67% baseline, 73% follow-up), Native American (84% baseline, 86% follow-up), and California Native (77% baseline, 73% follow-up), with a mean age of 45 years at baseline and 46 years at follow-up. The majority had an associate degree or higher (66% baseline, 64% follow-up). Geographically, over half were from Northern California (58% baseline, 54% follow-up). Only a small proportion of respondents reported using commercial tobacco products in the past 30 days (5% at baseline; 9% at follow-up), while approximately one-third reported lifetime use (33% at baseline; 38% at follow-up).Out of 86 baseline respondents, 23 of them (27%) were either project directors or project coordinators, while the remaining were community members. At follow-up, 19 of the 56 respondents (34%) were project staff.

**Table 1:** Survey participants by demographic and group characteristics

	Baseline	Follow-up
Gender (female)	58 (67%)	41 (73%)
Native American	72 (84%)	48 (86%)
California native	66 (77%)	41 (73%)
Average age	45.0	46.1
Education (associates or higher)	57 (66%)	36 (64%)
Lifetime commercial tobacco use (≥ 100 cigarettes)	28 (33%)	21 (38%)
Current commercial tobacco use (past 30 days)	4 (5%)	5 (9%)
Role (staff)	23 (27%)	19 (34%)
Org type (tribal government)	53 (62%)	36 (64%)
Region (north)	50 (58%)	30 (54%)
Region (south)	24 (28%)	16 (39%)
*Total respondents*	*n* = *86*	*n* = *56*

Analysis of the survey data commenced with descriptive methods to discern patterns, employing medians for Likert scale responses. Comparative analysis between baseline and follow-up data utilized Wilcoxon Rank-Sum Tests for Likert scale responses due to their non-parametric nature, to evaluate the changes in distributions over time. Further analytical steps included robustness checks encompassing assumptions testing, effect size analysis, and paired sample Wilcoxon Signed Rank Tests. Effect sizes were calculated using Cliff’s Delta, when appropriate, to determine the magnitude of changes.

### Interviews

A subset of respondents from the key informant survey, selected by the Project Directors, participated in 103 interviews conducted via the Zoom platform, with participants dialing in by phone. Each interview involved two CAITIE team members, an interviewer leading the dialog and a notetaker documenting the conversation and offering supplementary questions as needed. Interviews were conducted within one week of survey completion and typically lasted about 1 h, with a duration ranging from 30 min to 2 h. Interviews began with introductions, an overview of participant rights, and a consent request for recording. The interview questions then explored areas such as perceptions of the project’s impact, the partnerships and relationships developed by the project, community awareness of commercial tobacco risks, and community support for policy changes. Interviews were initially transcribed using the automated transcription software, Otter.AI, then verified for accuracy by at least two staff members, before being uploaded into Dedoose for analysis. Each transcript was reviewed by at least one Native American staff member, when possible.

Data analysis involved multiple rounds of qualitative coding. Initially, open coding was employed with three specific techniques: Descriptive, InVivo, and Values Coding [11]. Additionally, coders prepared coding memos throughout the first coding phase to refine the codes that encapsulated the identified patterns. Following the initial coding, a workshop was convened to synthesize findings and construct a unified codebook, via consensus, for subsequent coding phases. The team undertook a second coding round using the established codebook on baseline data, followed by a workshop to outline major themes and draft preliminary findings. As follow-up interviews were completed and transcribed, staff members were re-assigned transcripts for coding. A concluding workshop was organized to reconcile any coding discrepancies and finalize the key informant interview outcomes. The themes outlined in this article are the result of these workshops and reflect the opinions and experiences of the majority of respondents.

### Talking Circles

Talking circles were used to gather stories on the perceptions, questions, suggestions and understanding about commercial tobacco control efforts in communities served by initiative grantees. While originally tasked with conducting focus groups, the evaluation team turned to talking circles instead as a more culturally relevant approach. Although conceptually similar, talking circles are different from focus groups in their process and their outcomes [12–15]. Traditional talking circles have no fixed time limit, for example, and include opening and closing ceremonies, as well as a shared meal. Furthermore, the purpose of talking circles is to develop knowledge that is actionable and supports the collective well-being and survival of the Tribe [12]. Thus, while the data that comes from focus groups is often a transcript of what was discussed, the data from talking circles is the consensus achieved through that discussion.

Six talking circles with a total of 44 participants were conducted between May 2023 and January 2024 for this evaluation, three in-person and three virtually. As with the surveys and interviews, talking circle participants were identified by the Project Directors. The talking circles began with an opening ceremony led either by a Native American CAITIE staff member, or an appropriate community leader. The topics discussed included community perceptions of commercial tobacco use, changes observed since the beginning of the grant period, challenges to reducing commercial tobacco use, and aspirational questions on what they would like to see regarding commercial tobacco use in their community. In addition to these discussions, the CAITIE staff performed member checks with participants, sharing preliminary findings based on survey and interview data and requesting feedback to ensure their accuracy [16–18]. In-person talking circles also included a break for food.

In addition to the moderator, a CAITIE staff member served the role of support person and was available to help recommend questions and otherwise encourage the discussion. At least one additional CAITIE staff member was present for each talking circle to take notes throughout the discussion. Recordings were not made of the discussions, as that was deemed inappropriate and culturally insensitive. Given the nature of talking circles, notes were not taken with the goal of identifying individuals and their distinct opinions. Instead, the goal of the notes was to reveal the consensus reached in response to the questions we posed. After the talking circles ended, notes were gathered and used to write a narrative that explained the community’s position regarding our questions. Unique perspectives, stories, and direct quotes were included where appropriate and necessary to explicate a response but were otherwise de-emphasized.

## Results

Significant improvements were demonstrated between the two time points of data collection in the areas of community engagement, policy adoption and adherence, and Tribal capacity.

### Community Engagement

Community engagement was a key component to promoting commercial tobacco prevention in Tribal communities. Funded projects sought to build relationships with the Tribal community and did so by providing commercial tobacco awareness education and messaging, and by becoming a community resource for commercial tobacco prevention and other commercial tobacco-related health concerns. As a project staff member explained during an interview,

“I think [the success of the project has been] greater awareness and far more messaging than I’ve ever seen around here. You know, I think the more you hear something, the more you think about it. Getting people to change behaviors, that’s a real tough battle. But with the amount of—every gathering, every event there seems to be that message, every publication that we put out [has that message].”

**Fig. 1:**
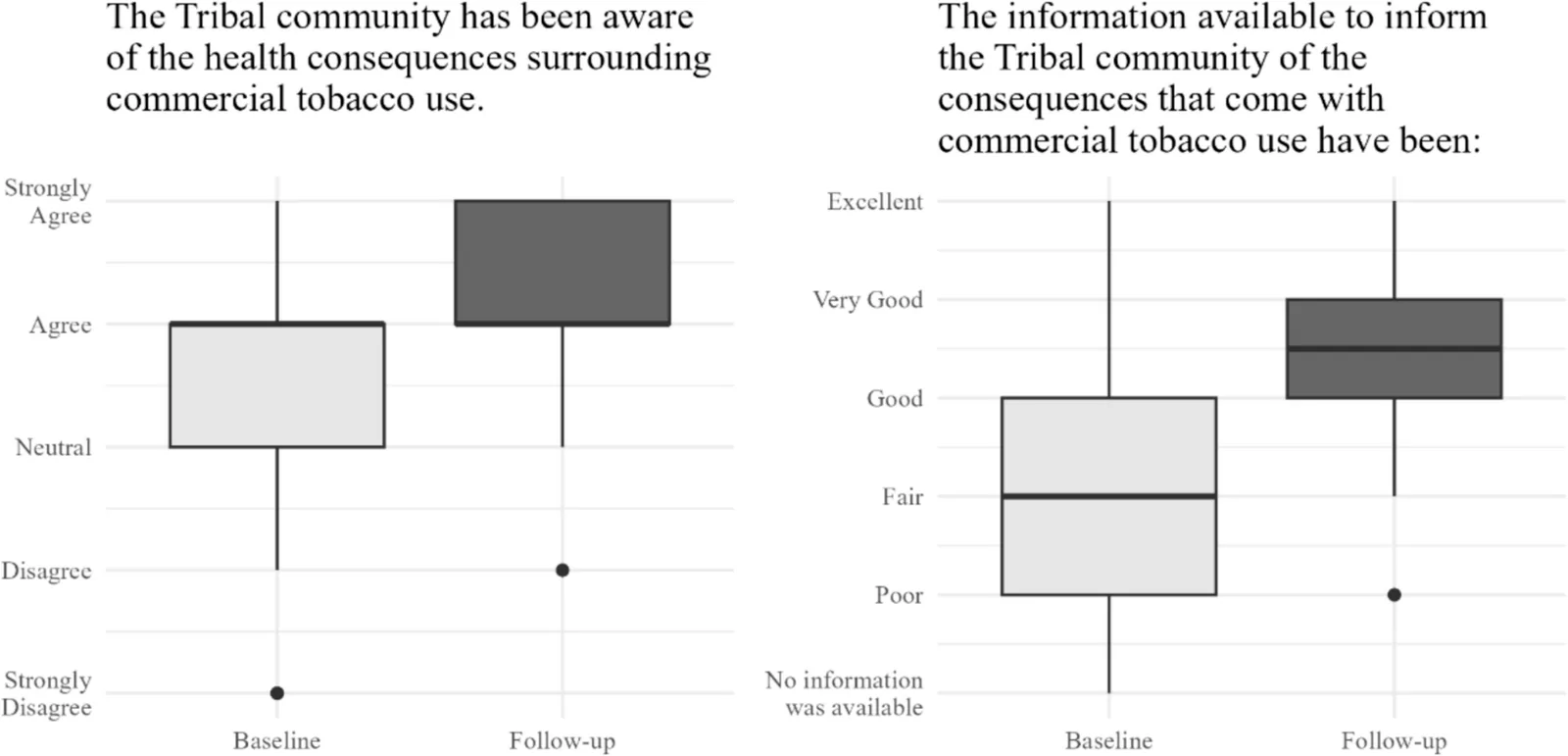
Changes in perceived community engagement over time

Improvements in community engagement were operationalized as perceived changes in the availability of information on harms associated with commercial tobacco use and the perceived community knowledge of associated harms. Survey responses showed improvements in both indicators, with positive and statistically significant changes (*p* < 0.001) (Fig. 1). Responses about the availability of information on the harms of commercial tobacco improved from a median of “Fair” or “Poor” to a median of “Good”. When asked if respondents believed that the Tribal community was aware of the harms associated with commercial tobacco use, the median remained consistent at “Agree” for both surveys. Statistical tests indicate positive and statistically significant changes in the distribution of both measures over time (*p* < 0.001) (Table 2). While the median for community awareness of harms did not change (4.0), the distribution of responses did change substantially, as reflected in the Cliff’s delta measure (0.344).

**Table 2:** Changes in perceived community engagement over time

	Baseline median (*n* = 86)	Follow-up median (*n* = 56)	Difference	*p*-value	Cliff’s delta (effect size)
Information	2.0	3.5	1.5	**0.000**	0.461
Awareness	4.0	4.0	0.0	**0.000**	0.344

Results are reported as the median answer to better reflect Likert style distributions

Median values reflect a range from 1 to 5, from negative to positive (disagree to agree)

The *p*-value is estimated using a Wilcoxon rank-sum test, appropriate for this data structure

The effect size is estimated by using Cliff’s delta. Positive values indicate a positive change in the distribution of responses

Bold values indicate statistically significant differences at *α* < 0.05

Youth engagement was a key component of community engagement for almost all American Indian Initiative grantee projects, regardless of whether it was written into their scope of work. Key Informants (KIs) emphasized that programming should focus on youth because it is easier to prevent commercial tobacco use in the first place rather than trying to encourage cessation of a highly addictive substance. Youth are also powerful messengers for normative change in the community. KIs shared that youth who attended their projects would express care to their parents, for example, by reminding them that they do not want them to smoke or vape because of the dangers to their health. KIs also said they witnessed youth reminding their friends and peers about the dangers of vaping and that they should not use those commercial tobacco products. As a community member explained,

“What I see working is the younger generation, the younger kids feel they have a voice now. When they see somebody smoking, and they’re too close to [a] building, they’ll tell them, ‘Can you read? We have signs up on the building.’ … And basically, I’ve witnessed them have them put that [cigarette] out. And stop smoking. And that was powerful to me to witness that ...[They’re] empowering those kids to take care of themselves better. Which they are, and they’re speaking up.”

Youth engagement is impactful immediately and through the generational transformation that it encourages. KIs expressed that youth, through being involved in programming, felt they had a voice in their community and were more vocal about the need for commercial tobacco control policies. Through some projects, youth were also given the opportunity to speak with Tribal council, elected officials in their counties, and politicians at the State Capitol about issues in their community related to commercial tobacco. This opportunity was seen as extremely transformative for the youth and for the community. As participants in a talking circle explained when asked about the role of collaboration in commercial tobacco prevention,

"All the knowledge about the problems with commercial tobacco could be taught to one or a few kids that are good speakers, who could then bring this up to the council. They can say, ‘we are the future. You are dealing with the now, but we need to implement these things for our future’. The collaboration could go further, with other Tribal communities in the area doing the same thing or working together on a group of young kids to do this work. These could be high school kids, articulate and with all the knowledge. If a peer goes up to them, they’ll just say ‘you can’t tell me what to do,’ but they can’t say that to kids. Sometimes, kids have to become the head man, in some cases, taking over the parenting role."

This theme of youth taking on leadership roles appeared again and again throughout the evaluation research. Youth are seen as the best targets for commercial tobacco prevention work, because prevention is perceived to be an easier goal than cessation, but they are also seen as the sources of that change. Commercial tobacco prevention programming should educate youth, but it should also empower them to bring about community-wide change.

### Policy Adoption

During the study period, Tribes adopted and implemented a range of policies to address commercial tobacco use in a variety of ways ranging from smoke-free health clinics and casinos to commercial tobacco sales to minors and tobacco taxes, among others (Table 3). Respondents reported significantly higher satisfaction rates with Tribal policies to reduce commercial tobacco use when comparing baseline and follow-up survey responses, and an overall higher perceived compliance rate across all policy types when comparing baseline to follow-up. When asked about their general satisfaction with the policy initiatives, the median response went from “Poor” in the baseline survey to “Good” in the follow-up, a substantial difference that was confirmed statistically (*p* < 0.001).

**Table 3:** Perceived median policy compliance rates over time

	Baseline median (*n* = 86)	Follow-up Median (*n* = 56)	*p*-value	Cliff’s delta (effect size)
Overall policy satisfaction	1.0	3.0	**0.000**	0.396
Multi-unit housing	1.0	2.0	**0.007**	0.292
Outdoor dining	2.0	3.0	**0.028**	0.231
Outdoor recreation	2.0	2.0	**0.012**	0.257
Smoke-free Health clinics	3.0	4.0	0.089	0.163
Within 20ft of entry	2.0	3.0	**0.000**	0.393
Workplaces	3.0	3.0	**0.009**	0.260
Smokeless tobacco	1.0	2.0	**0.006**	0.316
Sales age	2.0	3.0	0.126	0.169
Tobacco tax	2.0	3.0	**0.047**	0.265
Cessation	1.0	2.0	**0.004**	0.344
Litter	1.0	2.0	**0.003**	0.323
Smoke-free casinos	1.0	2.0	0.075	0.183

Results are reported as the median answer to better reflect Likert distributions

Numbers reflect a range from 1 to 5, from negative to positive (disagree to agree)

The *p*-value is estimated using a Wilcoxon rank-sum test, appropriate for this data structure

The effect size is estimated by using Cliff’s delta. Positive values indicate a positive change in the distribution

Bold values indicate statistically significant differences at *α* < 0.05

These results are further confirmed when examining perceived compliance rates for specific policies, with positive changes reported for almost every policy type. Respondents completed a matrix table rating compliance with various commercial tobacco policies. Policy types were listed as rows, and response options ranged from “No Policy or Program” to “Excellent” in answer to the question, “How would you rate compliance with the following commercial tobacco policies?” A statistically significant improvement from baseline to follow-up (*p* < 0.05) was observed for every policy area except *smoke-free Tribal health clinics*, *commercial tobacco sales age enforcement*, and *smoke-free casinos and gaming areas* (Table 3). The *smoke-free Tribal health clinics* and *commercial tobacco sales age enforcement* policies both already exhibited high perceived compliance, resulting in little change over time, whereas *smoke-free casinos and gaming areas*, despite their low perceived compliance rates, did not show improvement over time.

The policies with the most significant change in the distribution of responses over time (as reflected in the Cliff’s delta score) were found for *smoke-free within 20 or more feet of entryways of buildings*, *commercial tobacco cessation support*, *commercial tobacco litter/waste policies*, and *smokeless tobacco use policies*. While these policies exhibited the largest shifts over time, they were not necessarily perceived as the most common or successful by respondents. The policies with the highest perceived compliance rates were *smoke-free Tribal health clinics*, *tobacco tax*, c*ommercial tobacco sales age enforcement*, and *smoke-free workplaces*. The policies with the lowest perceived compliance rates were *smoke-free multi-unit housing*, *smoke-free outdoor recreation areas*, *smokeless tobacco use policies*, and *smoke-free casinos and gaming areas*, potentially due to infrequent implementation or lower adherence.

We conducted additional robustness checks to ensure that the observed changes in policy compliance rates were genuine and not merely a result of differences in survey respondents over time. When running the same analysis on a paired sample, limiting it to those who answered both the baseline and follow-up surveys, many of the observed effects were no longer statistically significant. Specifically, in the paired sample analysis, statistically significant changes were observed only for: *smoke-free multi-unit housing*, *smoke-free policies within 20 feet of building entryways*, *smoke-free workplaces*, and *commercial tobacco cessation support* (Table 4)*.*

**Table 4:** Perceived median policy compliance rates over time, paired sample analysis

	Baseline	Follow-up	*p*-value	Sample
Overall satisfaction	1.0	3.0	**0.002**	41
Multi-unit housing	1.0	2.0	**0.046**	33
Outdoor dining	2.0	3.0	0.134	35
Outdoor recreation	2.0	2.0	0.090	39
Health clinics	3.0	4.0	0.234	39
Within 20ft of entry	2.0	3.0	**0.009**	40
Workplaces	3.0	3.0	**0.003**	39
Smokeless tobacco	1.0	2.0	0.116	30
Sales age	2.0	3.0	0.097	32
Tobacco tax	2.0	3.0	0.075	23
Cessation	1.0	2.0	**0.006**	28
Litter	1.0	2.0	0.096	34
Casinos	1.0	2.0	0.423	36

Results are reported as the median answer to better reflect Likert distributions

Numbers reflect a range from 1 to 5, from negative to positive (disagree to agree)

The *p*-value is estimated using a Wilcoxon signed-rank test, appropriate for this data structure

Bold values indicate statistically significant differences at *α* < 0.05

The differences between the two analyses are likely due to the substantially smaller sample size in the paired analysis, making it more difficult to verify smaller effect sizes. Despite the smaller sample size, however, the observed change in overall satisfaction remained strong and statistically significant even when limited to only those respondents that answered both surveys. While these analyses raise questions about changes in certain policy areas, they overall support the conclusion that policy compliance improved from baseline to follow-up.

In both analyses, *Smoke-free casino* policies had the lowest median perceived adherence and did not see any significant change over time. Several Tribes in California do have smoke-free casinos, but threats to casino revenue are often seen as existential threats to some Tribal governments. The lack of significant progress on these policies is not surprising, and it may have an impact on the effectiveness of other commercial tobacco prevention programming. As community members explained during a talking circle,

"To me, it’s kind of a contradiction. The tribe has these tobacco grants to reduce smoking, but we have these casinos and getting revenue from tobacco sales. So yes, there is a concern from the community, but at the same time they are keeping tobacco around for the revenue. Commercial tobacco is normalized by their smoke shops and their smoke-friendly casinos. This sends mixed messages to the youth. How can you say it’s bad when it’s everywhere on the rez?"

Similar concerns were voiced by community members during talking circles and interviews with regard to policies limiting the sale of commercial tobacco more generally. Policies that could potentially hurt the economy of the Tribe were often perceived as off-limits. While some Tribes have been able to make changes on, for example, flavored tobacco sales, Tribes that rely on commercial tobacco revenue for their government are understandably wary to implement changes that might impact that revenue.

### Tribe Capacity Building

A key aim of the American Indian Initiative was to increase Tribal capacity to address commercial tobacco use. One way that capacity was built was through facilitating partnerships and relationships among and between Tribes and between Tribes and non-Tribal organizations. These partnerships and relationships played a significant role in individual project successes over the grant tenure. Projects developed important partnerships within their community and collaborative relationships with other grantees, which helped build capacity and improve programming. As a project director who had recently taken on the role explained,

“The connection is there. We work with all the neighboring Tribes, and then some other places that aren’t, you know, Tribal. So the connection is there. It’s been huge for me in a lot of areas that I’m not 100% familiar with...I have the people in place that I need in regard to—if I have a question or I need some feedback on this or whatever...I know the people to reach out to now.”

Several Tribes also worked together independently to develop a regional coalition that would collaborate on events and support each other as they fulfilled the grant objectives. This coalition turned out to be fundamental to the success of these projects, allowing them to fulfill their objectives together and to share ideas that became concrete action. As representatives from these projects explained during a talking circle when asked about the success of the initiative,

*The central achievement that was discussed was the coalition that was established by the Tribes in attendance. The coalition, started by one of the staff, was built from nothing and has become central to their work. They decided that they were going to support each other in their work for everything from questions on reporting, to figuring out how get events or activities funded, to holding events together like a collaborative youth summit. The person who brought the idea up originally added*, “The support that we’ve felt across the board from our coalition partners has really made change, and sustainable change, and it has made it joyful. It is our community. And our [...] community is strong.”

**Fig. 2:**
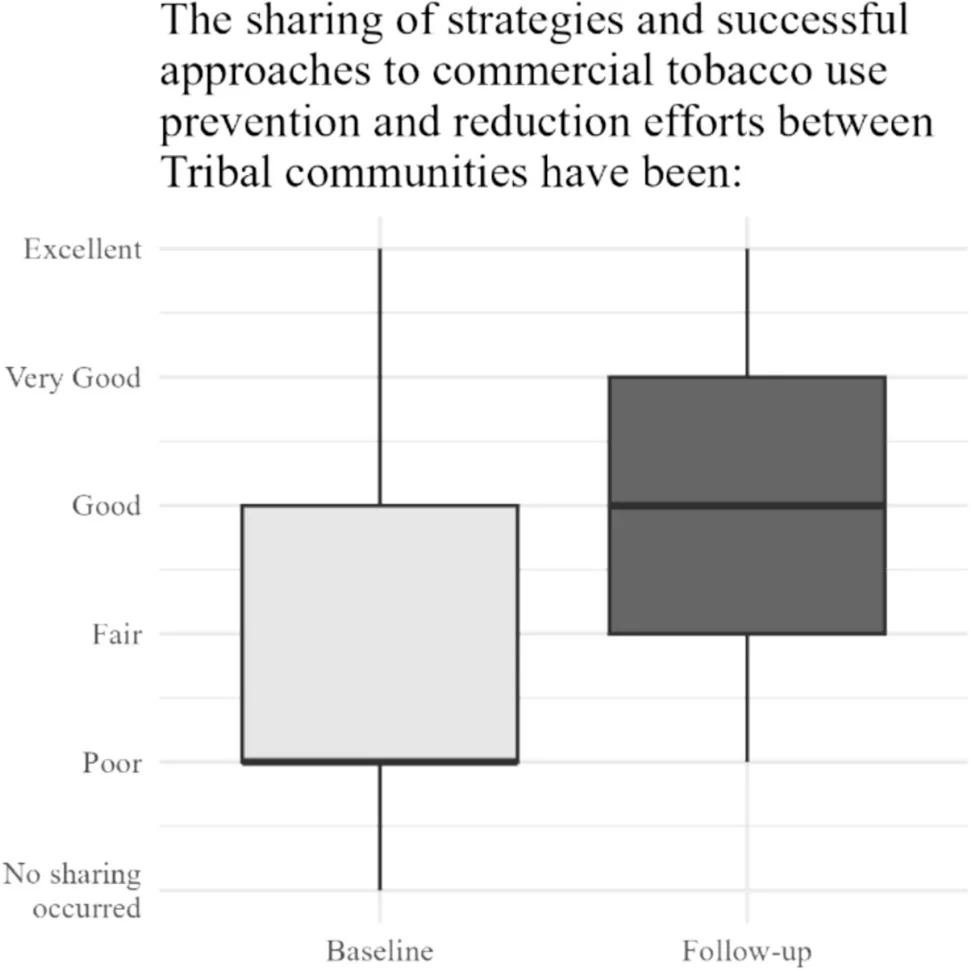
Satisfaction with how strategies and approaches were shared

In the survey, Tribal capacity was operationalized as the development of relationships within and between Tribes, as well as the reported satisfaction with available resources and the program’s sustainability. When asked to rate their satisfaction with how strategies and approaches were shared between Tribal communities in the survey, median responses changed from “Poor” in the baseline to “Good” in the follow-up, a substantial change that was statistically significant (*p* < 0.001) (Fig. 2).

The difference between the baseline and follow-up on this question was the largest effect we saw in the survey analysis, with an estimated effect size of 0.636, as estimated by Cliff’s delta. The shift came from respondents answering that there was no sharing (22% in the baseline to 0% in the follow-up) or that it was poor (37% in the baseline to 2% in the follow-up). We see a similarly dramatic shift from those that answered Excellent in the baseline (3%) to the follow-up (23%).

**Fig. 3:**
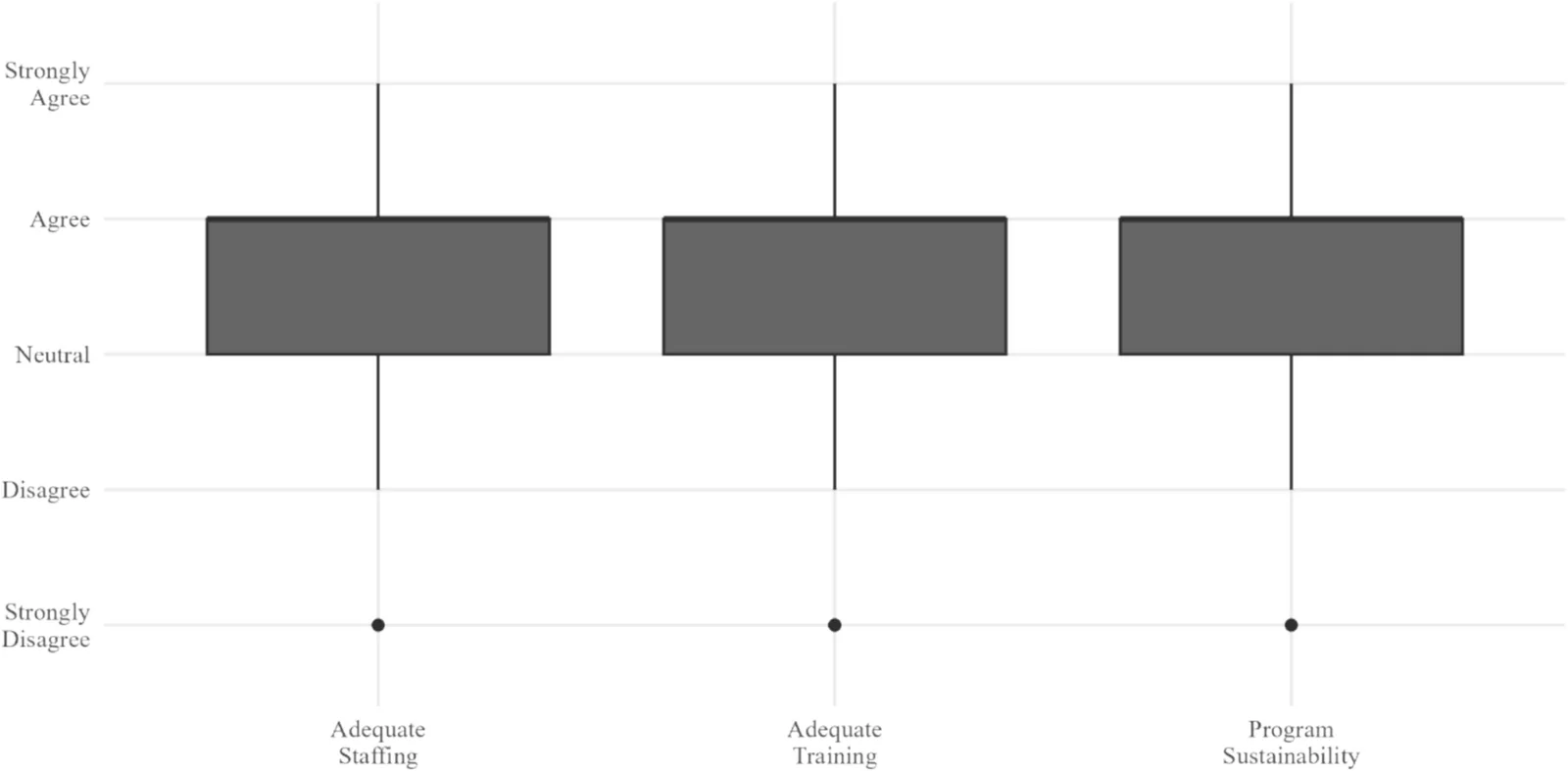
Satisfaction with staffing, training, and program sustainability

To assess perceptions of internal capacity building, follow-up survey respondents were asked to rate their agreement with statements about adequate staffing, available training, and program sustainability (Fig. 3). Median responses to all three items were “Agree”, suggesting overall satisfaction with the program’s internal implementation and the capacity built through the initiative.

In interviews, staff KIs shared that project success needed to be measured in stages; the current stage, for many projects, was building up capacity, awareness, and resources that were deeply rooted in the community and were responsive to the community’s specific needs. As this was the first time that Tribes were individually funded for commercial tobacco control and prevention, there was significant foundational work that needed to be done in most projects. As a community engagement staff member explained,

“My personal strategy, and it’s one that seems in alignment with my project director, is one of building: building education, building awareness, building collaboration, building community buy-in, building community engagement. But from my experience in community those are long processes in Tribal communities. Those are long and very, like, intimate personal processes, as well.”

Staff echoed this sentiment during a talking circle, emphasizing the importance of developing relationships and a presence in the community, and this is especially true when staff are not from the communities they serve.

*When asked about the change they’ve seen in the community since the start of the program, one person answered,* “That’s an easy one, we have a voice. The community knows who we are, and they are expected to be at all the events. If any Tribal event is held, the community know that Tobacco’s gonna show up.” *The success of the programs has not come easily or quickly, however. All staff present on the call were not from the Tribal communities they work with, so they had to build up that rapport and that trust. But now they have established that relationship, and that means that,* “When you go out wherever, they recognize you. And once they realize who we are, then they know we don’t pose a threat. And then we are certainly welcomed.”

To better understand the successes in capacity building achieved by grantees, the follow-up survey included additional questions on the resources the project had to achieve its goals and its perceived sustainability beyond the limits of the grant. Responses to these questions were, overall, very positive. When asked about staffing, for example, 63% of respondents in the follow-up survey either agreed (47%) or strongly agreed (16%) that the project had been adequately staffed. Respondents were similarly optimistic when asked about the sustainability of the project, with only 8% either disagreeing (4%) or strongly disagreeing (4%) that the project has the ability to sustain itself in the future. Finally, when asked if the project staff had the training and technical support that it needed to run a successful project, 71% either agreed (50%) or strongly agreed (21%). One KI summed up success for them by saying, “it’s not perfection, but progress.”

## Discussion

The results from this study provide evidence for funding Tribal governments and local community organizations to do the work of culturally relevant commercial tobacco prevention. Specifically, the study results suggest that this funding structure is an effective strategy for (1) engaging the community in commercial tobacco prevention, (2) passing policies related to commercial tobacco prevention, and for (3) building the local capacity necessary for communities to continue to do this work. The initiative structure was able to do this by respecting and elevating local knowledge and community members, who know best what their community needs and how to push for that change [19].

The level of community engagement with the commercial tobacco prevention programs was strongly impacted by the grant funding structure. The results showed a substantial, positive, and statistically significant improvement in the community’s awareness of harms associated with commercial tobacco use, as well as the quality of information that was available on commercial tobacco. This suggests that the projects were able to reach community members and encourage them to engage with their events and initiatives. Interview and talking circle data further confirm the importance of locally relevant materials. Staff interviewees emphasized, and community interviewees confirmed, for example, that participants were most engaged when they taught about local traditional tobacco practices and how they are different from commercial tobacco use, as well as the history of the commercial tobacco industry’s intentional targeting of American Indian communities. This difference in engagement was particularly stark for youth who may otherwise have been uninterested in learning about the harms of commercial tobacco.

Passing policy on commercial tobacco prevention in Tribal communities is a difficult process mired with logistical, economic, and cultural barriers. In a study of tribal communities in California, for example, Soto et al. found that respondents were largely supportive of what they called “small-p” policies like putting up non-smoking signs, but they were opposed to “big-P” policies like smoke-free casinos and smoke-free housing [20]. Unlike “small-p” policies, the “big-P” policies were largely rejected as threats to the ability of the Tribe to sustain itself economically [20]. The substantial, positive, and statistically significant difference in overall policy adherence from baseline to follow-up observed in this study, including big-P policies like support for a *tobacco tax*, *smoke-free outdoor dining*, and *smoke-free multi-unit housing*, suggests that what was done by the American Indian Initiative overcame this reticence, at least to some extent. The overall low support for *smoke-free casinos,* however, was replicated in our findings.

The most effective consequence of locally relevant programming, according to our results, might be the Tribal capacity that is built through local funding and the support for local knowledge and customs. The strong change in participants’ perceptions of the quality and quantity of information and strategies shared between Tribes, in particular, emphasizes the consequent development of community capacity to do this work. Relationships are fundamental to work in American Indian communities, and this initiative was able to bolster those relationships to a significant degree [21, 22]. These results also replicate findings from other studies on the sustainability of Native American programming, including on the sustainability of suicide prevention programs [23]. Finally, funding Tribal governments and American Indian community organizations directly to do this work contributes to the development of what Chino and Lemyra called the “true capacity” for public health programming in American Indian communities [24]. Such a capacity draws explicitly from local and diverse Indigenous ways of knowing and is based on “Tribal communities […] taking control of their own health services and health promotion efforts” [24].

### Study Challenges and Limitations

The study relied on self-reported data to gauge outcomes such as commercial tobacco use rates, policy adoption rates, and community engagement. Consequently, these metrics depend on key informant perceptions rather than empirical counts, which may not fully capture the actual rates of commercial tobacco use, policy adoption, or community engagement.

Furthermore, the agreement between the CDPH/CTPP and the participating Tribal governments assigned the responsibility for participant recruitment to project directors. This decision respected each Tribe’s sovereign right to control data shared with the state of California. However, this arrangement could potentially affect the validity of our research. Project staff, potentially motivated by a desire to present the project favorably, could influence the outcomes. Comparative analysis of responses by staff and community members showed that staff members often reported more pessimistically on success metrics such as policy adoption and perceived smoking rates than community members. This suggests that the community members recruited by project directors may have been more positive toward the program than respondents selected through a different sampling process. At the same time, the candid responses of staff indicate a greater willingness to acknowledge challenges, countering assumptions that their perspectives would be overly positive.

Due to difficulties with outreach, as well as an expected amount of drop-off from baseline to follow-up, there was some data missing from the follow-up data collection. For surveys, the difference amounted to 30 respondents (35% of the baseline sample of 86), while for interviews, it amounted to 11 respondents (19% of the baseline sample of 57). This incongruency between baseline and follow-up may have influenced our results. Projects that had intermittent staffing or were otherwise difficult for the CAITIE team to engage may be systematically different from those communities that were easier to reach and schedule. However, the interview and survey samples included at least one respondent from every community during baseline and follow-up, so no Tribes were completely excluded.

## Conclusion

Despite broad reductions in commercial tobacco use nationally, health disparities persist between the general population and American Indians. The American Indian Initiative in California aimed to address these by directly funding Tribes to develop targeted commercial tobacco prevention and control programs. This effort, designed in collaboration with Tribal governments across the state, ensured that the initiative’s structure and goals were inclusive, relevant, and effective. The initiative focused on three main objectives: (1) enhancing community engagement in commercial tobacco systems change campaigns, (2) advocating for and implementing policies to reduce tobacco use, and (3) building Tribal capacity for long-term program sustainability. This study evaluated whether these objectives were met, and in so doing assessed the effectiveness of directly funding Tribes for public health programming. The results indicate significant, positive changes across these areas, affirming the value of this strategy. Feedback from staff and community members through surveys, interviews, and talking circles highlighted increased awareness of tobacco harms, support for prevention policies, and enhanced community capacity.

Project staff and community members consistently and repeatedly emphasized the importance of this work. Native American communities are disproportionally impacted by the commercial tobacco industry; they have been explicitly targeted, and their culture coopted to serve a foreign industry that impedes the health and longevity of communities. Change has not come fast enough, but it is coming. Projects like the American Indian Initiative serve as catalysts for this change, promoting healthier behaviors, supporting alternative coping mechanisms, and otherwise providing the economic support necessary for this work. The innovation of the American Indian Initiative in funding Tribal communities directly has the potential to support change where other approaches have been less successful. Moreover, this approach respects Tribal sovereignty and elevates Indigenous ways of knowing, both effectively and respectfully, increasing community engagement and ownership of the programming. By recognizing the diversity within Tribal communities and promoting their autonomy, this strategy not only advances public health but also supports the cultural and economic resilience of Native American Tribes.
